# Two Rare Variants of Down Syndrome: Down-Turner Syndrome and Down Syndrome with Translocation (13;14): A Case Report

**Published:** 2019-11

**Authors:** Evren GUMUS

**Affiliations:** Department of Medical Genetics, Faculty of Medicine, University of Harran, Sanliurfa, Turkey

**Keywords:** Down syndrome, Double aneuploidy, Translocation

## Abstract

In the present paper, we report two rare cases of Down syndrome (DS); mosaic Down-Turner syndrome and DS with rob (13;14). Patient 1 karyotype is mos 45,X[41] / 47, XX,+21[59] and patient 2 karyotype is 46, XY, rob (13;14)(q10;q10),+21. With these two unusual cases, we aimed to look at the most common numerical and structural chromosome anomalies from a different window and evaluate the phenotypic effect in the presence of different chromosomal anomalies. Our main goal is to evaluate the phenotypic characteristics of these two rare variants in the light of literature.

## Introduction

Down syndrome (DS), also known as trisomy 21, is a chromosomal disease caused by the existence of a third copy of the 21^st^ chromosome. DS is the most widespread chromosomal disease that we know of. The WHO noticed that the incidence of DS is from 1/1000 to 1/1100 live births. There are three main types of DS; trisomy 21 (most common form, all cells have a triple copy of 21^st^ chromosome), mosaicism (some cells have three copies of chromosome 21 and some cells have two copies of chromosome 21) and translocation (a third copy of chromosome 21 is translocated to another acrocentric chromosome) (1, 2). Apart from these main types, DS with sex chromosome aneuploidy and DS with translocation not including chromosome 21 can be observed. To date, DS together with many sex chromosome aneuploidies such as XXX, XXXY, XYY, XXY (Klinefelter) and monosomy X (Turner) have been identified. Simultaneous coexistence of DS and Klinefelter syndrome is relatively frequent.

On the contrary, the combination of DS and Turner syndrome is very rare (estimated frequency is 1 in 2,000,000) (3–7). Translocation type DS constitutes about 5% of all down syndromes but DS with translocation not including chromosome 21 is quite rare (1). In a study of 1223 individuals with DS, only one person with a translocation of chromosome 13 and 14 was observed (8).

In the present paper, we report a two very rare variants of DS; mosaic Down-Turner syndrome and DS with a robertsonian translocation between the two chromosomes 13 and 14. Our main goal is to evaluate the phenotypic characteristics of these two rare variants in the light of literature.

## Case Report

This study was conducted in accordance with the Declaration of Helsinki amended in 2013. Informed consent was obtained for the genetic analysis of the patient and parents, the publication of patient data and photos.

**Fig. 1:**
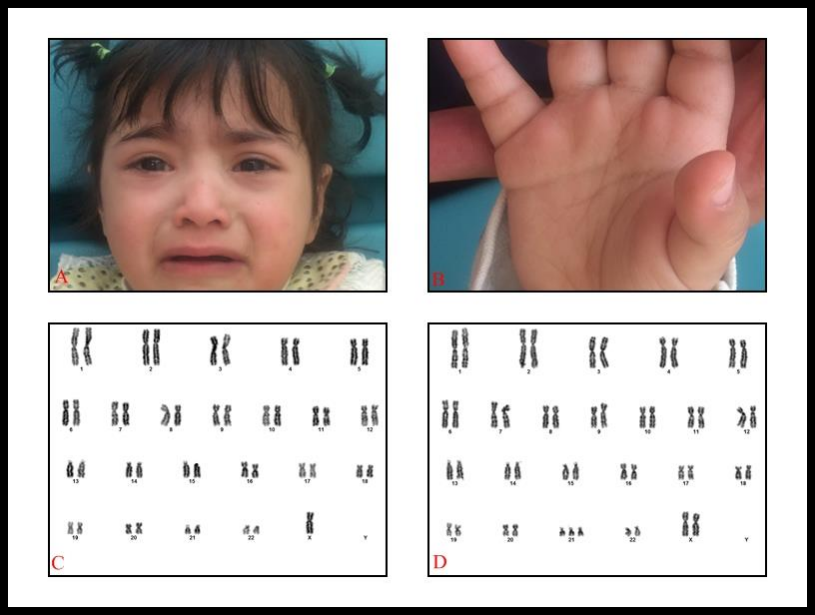
Phenotypic and dysmorphic features of patient 1 (A and B), karyotype of patient 1 (C and D)

Patient 1, a two-year old girl, first child of the family, born to healthy 26-year-old mother and 28-year-old father of Turkish origin. Data on antenatal research were not found. She had been born at term by spontaneous vaginal delivery without any complication. Her birth weight was 3100 gr (25–50 percentile) and birth length was 50 cm (50–75 percentile). Occipitofrontal circumferences (OFC) at birth is unknown. The patient is referred to our department for suspicion of DS. On the examination, her weight is 11 kg (25–50percentile), her length is 87 cm (50–75percentile) and her OFC is 48.5 cm (50–75 percentile). Biochemical tests of thyroid, kidney and liver function were normal. Ophthalmological examination and echocardiogram were normal. Ultrasound imaging showed hepatomegaly and renal hypoplasia. Blood count and peripheral smear were normal. Minor dysmorphic features such as brachycephaly, upslanting palpebral fissures, broad nasal base and epicanthus were noted. No single transverse palmar crease was observed. Karyotype from the peripheral lymphocytes using G-bandingis ‘mos 45,X[41] / 47, XX,+21[59]’ (Fig. 1). Karyotypes of parents were normal.

Patient 2, a three-month old boy, born to healthy 21-year-old mother and 24-year-old father. This was the mother’s first pregnancy. The information that no problems were detected in antenatal follow-ups was shared by the family. He had been born at term by spontaneous vaginal delivery with 6/7 Apgar index without any complication. Her birth weight was 3500 gr (50–75 percentile), birth length was 49 cm (25–50 percentile) and occipitofrontal circumferences (OFC) at birth was 33 cm (3–10 percentile). The patient is referred to our department for suspicion of DS.

**Fig. 2:**
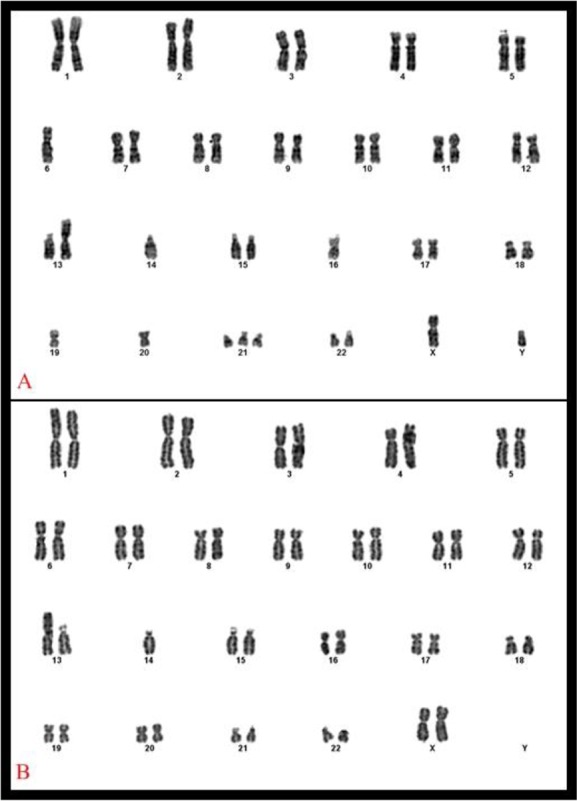
Karyotype of patient 2 (A), karyotype of patient’s mother (B)

On the examination, his weight, length and OFC were 25–50 percentile. Biochemical tests of thyroid, kidney and liver function were normal. Ophthalmological examination and echocardiogram were normal. Ultrasound imaging showed no abnormality. Blood count and peripheral smear were normal. Minor dysmorphic features such as brachycephaly, upslanting palpebral fissures and single transverse palmar crease were noted. Karyotype from the peripheral lymphocytes using G-banding was ’46, XY, rob (13;14)(q10;q10),+21’. Chromosomal karyotypes of parents were requested. Father’s karyotype was normal and mother’s karyotype was ‘45, XX, rob (13;14)(q10;q10)’ (Fig. 2). The maternal grandparents of the propositus were dead. Chromosomal microarray studies for the patient were performed with the Affymetrix CytoScan Optima (315k) (Thermo Fisher Scientific, MA, USA) chips from the DNA obtained from the peripheral blood. Analyzes were made according to the protocol of the manufacturer. All data were analyzed in the ChAS 3.1 program (Thermo Fisher Scientific, MA, USA). Microarray result showed no abnormality except trisomy 21.

## Discussion

Aneuploidy is defined as the abnormal number of chromosomes, which is basically associated with advanced maternal age and frequently observed, but double aneuploidy is a rare condition. Also, interestingly in the vast majority of double aneuploidy cases, mothers are under 35 years old. Double aneuploidy is often the result of one autosomal and one sex chromosome aneuploidy. It is usually seen as double trisomy. Double aneuploidy was first described in a patient with both Klinefelter syndrome and DS (9). Sex chromosome monosomy and autosomal trisomy are very rare. In double aneuploidy cases, at early ages, the clinical picture is completely compatible with the autosomal chromosome aneuploidy. This is mainly due to the observation of the phenotypic effects of sex chromosomal aneuploidies in the post pubertal period (2–4,10,11). In our patient 1, we observed many DS stigmata’s such as upslant palpebral fissures, brachycephaly and epicanthus. In addition to these findings, renal hypoplasia and hepatomegaly may be due to Down or Turner syndrome. For DS and Turner syndrome all the necessary examination of our patient was done but it should be remembered that Turner syndrome may become dominant in post pubertal period.

Translocation is a chromosomal abnormality caused by rearrangement of parts between nonhomologous chromosomes. Robertsonian translocation is centromeric fusion of wo acrocentric chromosomes, and most common Robertsonian translocation is between chromosome 13 and 14. The Robertsonian translocation carriers are usually healthy person but they have a reproductive risk for loss or gain of genetic material (12). Sometimes, translocation is transferred over generations and it is almost impossible to diagnose it unless there is any phenotypic effect. As long as there is no phenotypic effect and reproductive failure, it is unnecessary to do additional testing but it is different for our unusual patient because of the association of DS with a maternally transmitted Robertsonian translocation of chromosome 13 and 14. This is necessary so that the observed phenotypic effects are attributed to trisomy 21. For this reason, we requested a micro-array test from the patient. We did not detect any anomaly other than trisomy 21 in the result, so, we associated the findings with trisomy 21 with inner peace.

With these two unusual cases, we aimed to look at the most common numerical and structural chromosome anomalies from a different window and evaluate the phenotypic effect in the presence of different chromosomal anomalies. Prenatal diagnosis and genetic counseling for the next pregnancy was recommended for patients’ families.

## Ethical considerations

Ethical issues (Including plagiarism, informed consent, misconduct, data fabrication and/or falsification, double publication and/or submission, redundancy, etc.) have been completely observed by the authors.
